# Correction

**DOI:** 10.1080/15384047.2024.2352926

**Published:** 2024-05-15

**Article title: Temozolomide renders murine cancer cells susceptible to oncolytic adenovirus replication and oncolysis**

**Authors**: Rodolfo Garza-Morales, Kavitha Yaddanapudi, Rigoberto Perez-Hernandez, Eric Riedinger, Kelly M. McMasters, Haval Shirwan, Esma Yolcu, Roberto Montes de Oca-Luna, Jorge G. Gomez-Gutierrez

**Journal**: Cancer Biology and Therapy

**Bibliometrics**: Volume 19, Number 3, pages 188–197

**DOI**: https://doi.org/10.1080/15384047.2017.1416274

The authors recently noticed that in this article, the western blot of actin in Figure 2C center was inadvertently misplaced in Figure 3C left during the preparation of these figures. The amended version of these figures is now shown below. The conclusions of this paper are not affected. The authors sincerely apologize for this error.

**Figure 2.**
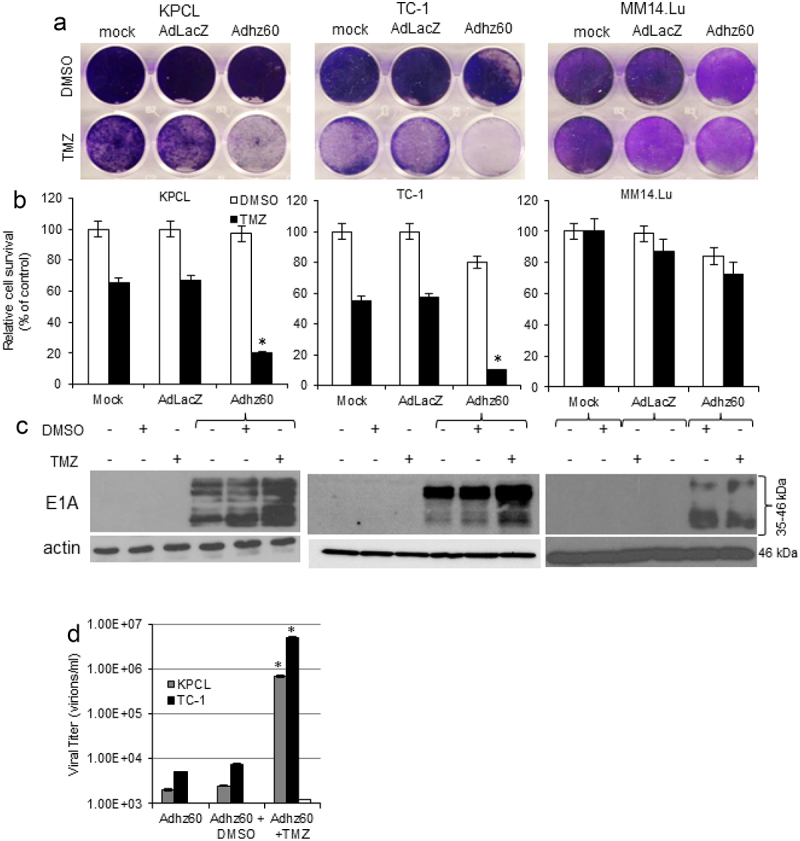
**Effect of combined therapy of TMZ and OAd on virus replication in murine cancer and non-cancerous cells. (A)** Murine cancer KPCL and TC-1 and non-cancerous MM14.Lu cells were treated with Adhz60 and TMZ at the following doses for Adhz60 and TMZ, respectively 10 MOI and 400 µM. AdLacZ was used at 10 MOI for all cell lines. DMSO was added as a control at its respective volume for each cell line. At 72h post-infection, crystal violet staining was used to evaluate CPE. A representative staining is shown of three experiments performed. **(B)** OAd-mediated CPE was calculated by measuring the absorbance of solubilized dye at 590 nm. Results represent the mean of three repeated measurements ± standard deviation (SD; *error bars*) (*P < 0.05 for all cell lines). **(C)** Expression of adenovirus E1A proteins were detected with an anti-adenovirus type 5 E1A monoclonal antibody. Actin was used as a loading control. A representative experiment is shown from three performed. **(D)** Supernatants from Figure 2A were used to determine adenovirus yield from each cell line. Results represent the mean of three independent experiments ± standard deviation (SD; error bars) (*P < 0.05).

**Figure 3.**
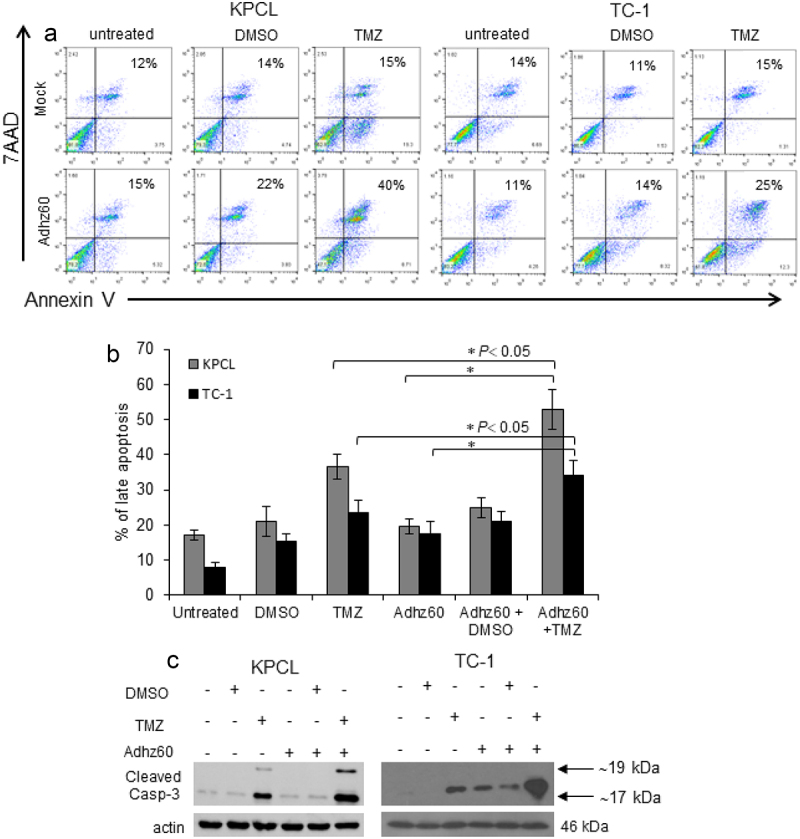
**Evaluation of Adhz60 and TMZ combined therapy-induced apoptosis**. Murine cancer cells were untreated or treated with DMSO, TMZ, and Adhz60-alone, or in combination as described in Figure 2. **(A)** At 72 h post-treatment, murine cancer cells were stained with annexin V-PE and 7-aminoactinomycin D (7-AAD). Positive cells for annexin V-PE and 7-AAD staining were analyzed by FACScan flow cytometer with FlowJo software. **(B)** Results represent the mean of three independent experiments ± standard deviation (SD; *error bars*) (*P < 0.05). **(C)** Whole cell protein lysates were collected 72h after indicated treatment. Expression of cleaved caspase-3 was detected by Western blotting. Actin was used as a loading control. A representative experiment is shown from three performed.

